# Supplementary material for: Cytoplasmic DAXX drives SQSTM1/p62 phase condensation to activate Nrf2-mediated stress response
Source: Nat Commun. 2019 Aug 21;10:3759. doi: 10.1038/s41467-019-11671-2 (PMC6704147; doi:10.1038/s41467-019-11671-2)
Supplement: Supplementary file 1 — Supplementary Information [file 41467_2019_11671_MOESM1_ESM.pdf]

Supplementary Information

Cytoplasmic DAXX drives SQSTM1/p62 phase condensation to activate Nrf2-mediated stress response

Yang et al.

Supplementary Figure 1

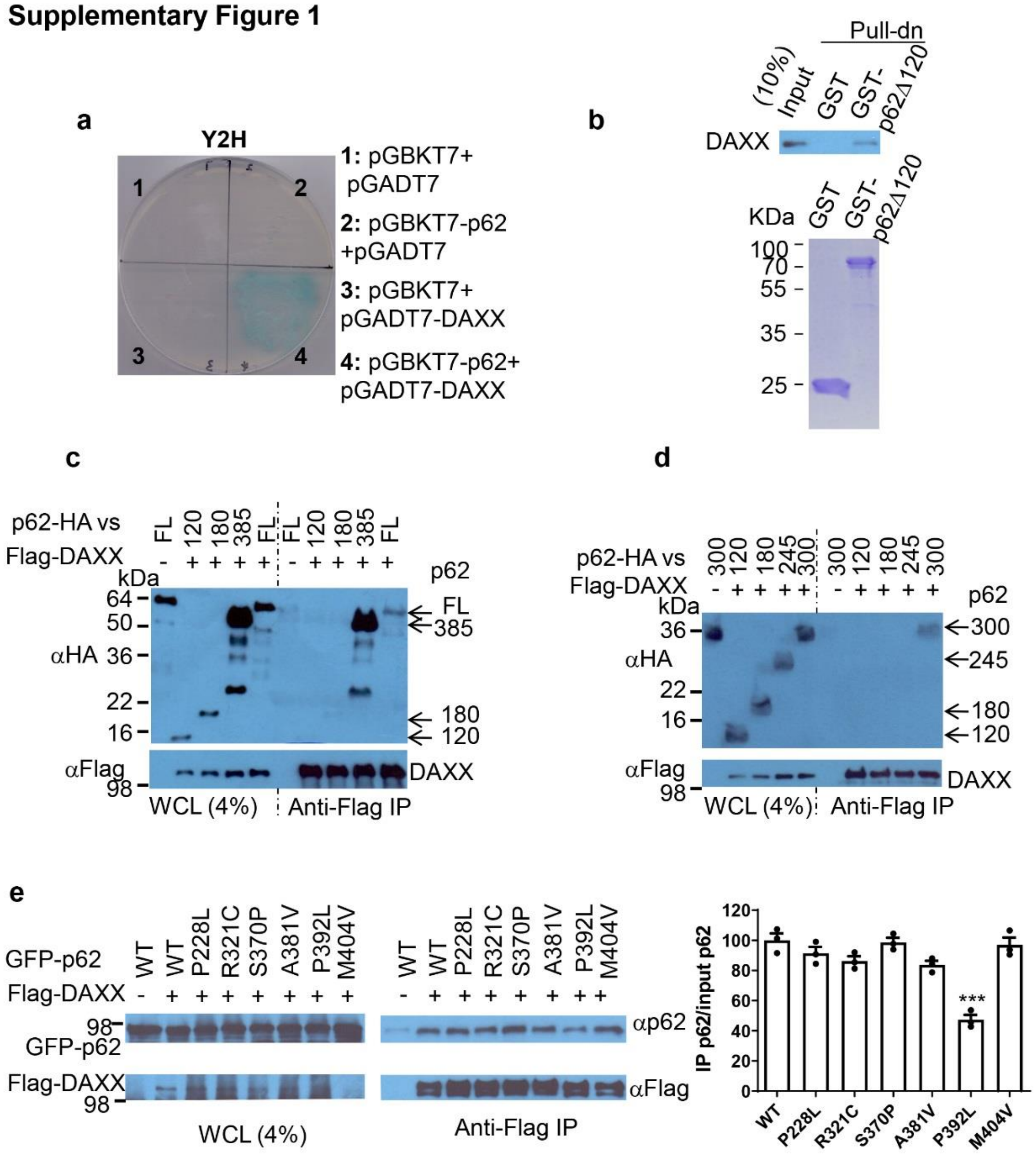

Supplementary Figure 1. To define the region in p62 required for DAXX interaction. (a) Confirmation of p62-DAXX interaction in yeasts by co-transformation of p62 and DAXX into yeasts. p62 was used as a bait to screen its binding proteins. DAXX was identified as a potential p62-interacting partner. Survival of yeasts in nutrient-deficient media by co-transformation of p62 and DAXX confirms the DAXX-p62 interaction in yeasts. **(b)** *In vitro* p62-DAXX interaction (pulldown by GST-p62 with 1-120aa deletion). *In vitro* translated DAXX (5 µl) was incubated with purified glutathione-bound GST or GST-p62Δ120 overnight. The pull-down (Pull-dn) products were subjected to SDS-PAGE and probed with anti-DAXX antibody. 0.5 µl of *in vitro* translated DAXX was used for input. **(c)** p62 1-180aa is not required for DAXX binding. p62-HA full-length (FL)/empty vector (for negative control), p62 1-120aa-HA/Flag-DAXX, p62 1-180aa-HA/Flag-DAXX, p62 1-385aa-HA/Flag-DAXX, or p62-HA-FL/Flag-DAXX were co-transfected into HeLa cells. After 20 hours, anti-Flag (M2)

was used for immunoprecipitation (IP). The immunoprecipitates and the whole cell lysates (WCL) were probed with anti-HA (mouse), and anti-Flag (rabbit). The dot lines separate the WCL and IP samples. vs: variants. **(d)** p62 246-300aa is required for DAXX binding. p62 1-300aa-HA (FL)/empty vector (for negative control), p62 1-120aa-HA/Flag-DAXX, p62 1-180aa-HA/Flag-DAXX, p62 1-245aa-HA/Flag-DAXX, p62 1-300aa-HA/Flag-DAXX were cotransfected into HeLa cells. After 20 hours, anti-Flag was used for IP. The immunoprecipitates and the WCL were probed with anti-HA (mouse), and anti-Flag (rabbit). vs: variants. (**e)** GFP-p62/empty vector (for negative control), GFP-p62/Flag-DAXX, or GFP-p62 mutants indicated/Flag-DAXX were cotransfected into HeLa cells. After 20 hours, anti-Flag was used for IP. The immunoprecipitates and the WCL were probed with anti-p62, and anti-Flag. The ratios of immunoprecipitated p62 versus input p62 were quantified. n=3 independent experiments. Statistical analysis was performed by one-way ANOVA. Tukey's test was used for the comparison. ***: $P<0.0001$. Data are shown as mean±sem.

Supplementary Figure 2

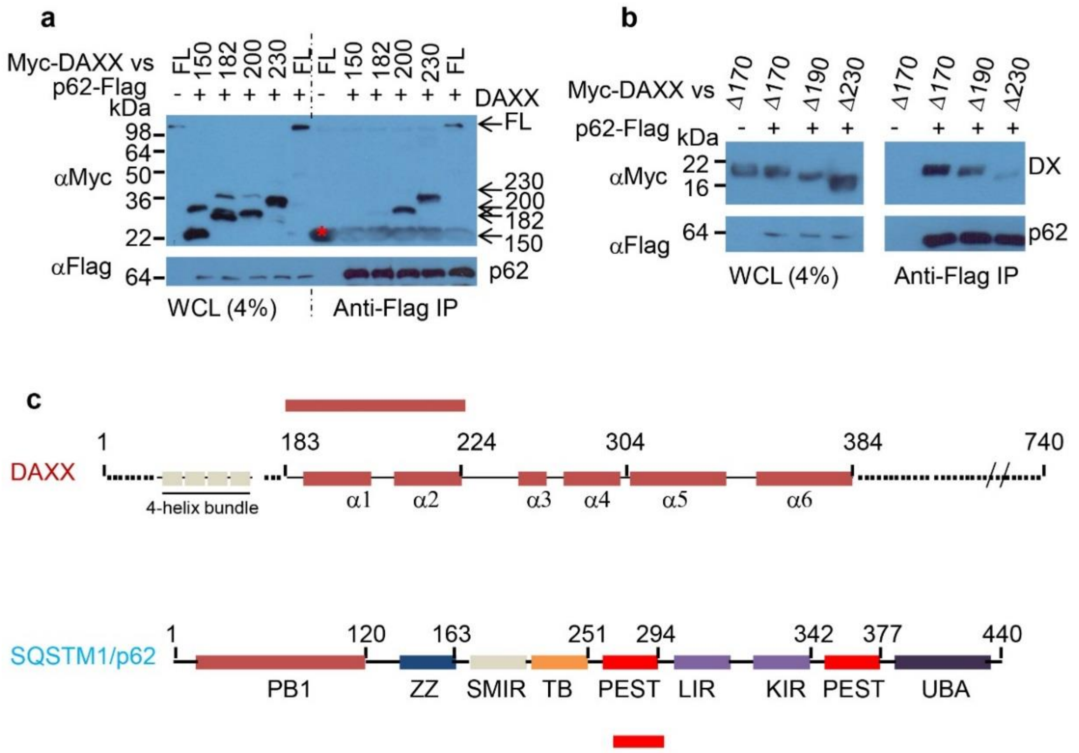

Supplementary Figure 2. To define the region in DAXX required for p62 interaction. (a) Myc-DAXX full-length (FL)/empty vector, Myc-DAXX 1-150aa/p62-Flag, Myc-DAXX 1-182aa/p62-Flag, Myc-DAXX 1-200aa/p62-Flag, Myc-DAXX 1-230aa/p62-Flag, or Myc-DAXX-FL/p62-Flag was cotransfected into HeLa cells. After 20 hours, anti-Flag (M2) was used for IP. The immunoprecipitates and the WCL were probed with anti-Myc (rabbit), and anti-Flag (rabbit). * denotes antibody light chain. **(b)** Myc-DAXX with 171-370aa (1-170 deletion, Δ170)/empty vector, Myc-DAXX 171-370aa (Δ170)/p62-Flag, Myc-DAXX 190-370aa (Δ190)/p62-Flag, or Myc-DAXX 231-370aa (Δ230)/p62-Flag was cotransfected into HeLa cells. After 20 hours, anti-Flag was used for IP. The immunoprecipitates and the WCL were detected with anti-Myc, and anti-Flag. DX: DAXX. (**c)** DAXX contains a 4-helix bundle at its amino terminus and a histone-binding domain (HBD) comprising 1-6 α-helices. The domains in p62. p62 has a Phox1 and Bem1p (PB1) domain, a zinc finger (ZZ) domain, an SOD mutant interaction region (SMIR), a tumor necrosis factor receptor-associate factor 6 (TRAF6)-binding (TB) domain, an LC3-interacting region (LIR), a Keap1-interacting region (KIR), 2 PEST domains that are rich in amino acids proline (P), glutamic acid (E), serine (S) and threonine (T), and a ubiquitin-associated (UBA) domain. PB1 promotes homo-oligomerisation as well as hetero-oligomerisation with other proteins including NBR1, aPKC, caspase-8 and ERK1. The ZZ domain interacts with RIP, and TB binds to TRAF6. p62 interactions with TRAF6, RIP and aPKC are involved in NF-kB activation. KIR mediates p62-Keap1 interaction, and LIR interacts with LC3. UBA binds polyubiquitin chains. The region between ZZ and TB binds to Raptor, a subunit of mTORC1 complex.

Supplementary Figure 3

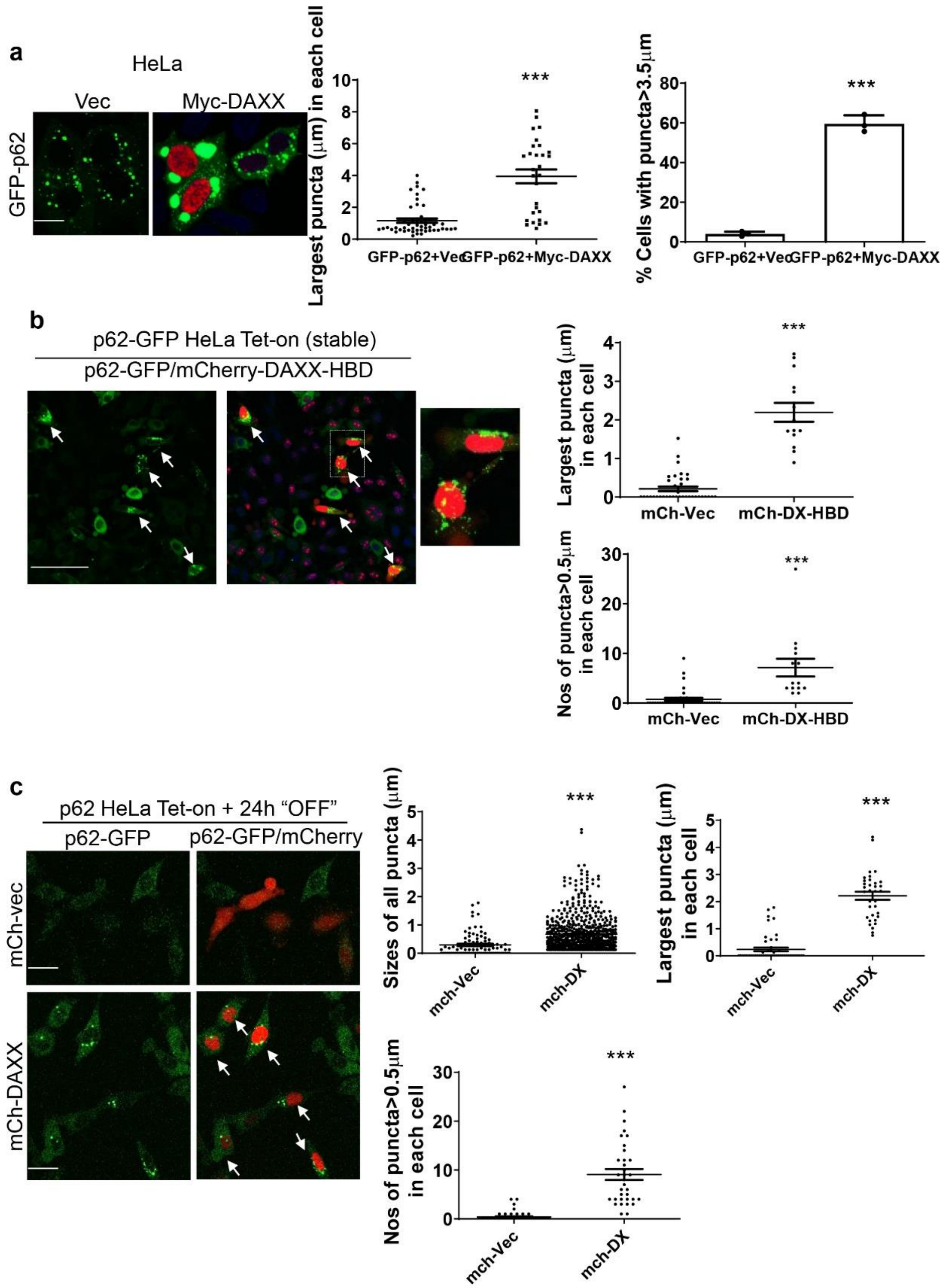

Supplementary Figure 3. DAXX promotes p62 body formation. (a) GFP-p62/vector or GFP-p62/Myc-DAXX plasmid were co-transfected into HeLa cells. After 20 hours, the cells were fixed, and stained with anti-Myc antibody. The images were acquired with a confocal microscope. Bar: 10 μm. The diameter of the largest GFP-p62 puncta (μm) in each cell was measured with LAS AF Lite. n=50, 29 cells. The percentage of

the cells with puncta >3.5 µm was graphed. n=3 biological replicates. Statistical analysis was performed with T-tests. ***: P<0.0001. **(b)** HeLa Tet-on cells stably harboring p62-GFP were transfected with mCherry vector or mCherry-DAXX H3.3-binding domain (HBD, 178-417aa). 3 hours after the transfection, Doxycycline (Dox) was added to HeLa Tet-on cells to induce p62-GFP expression. After further 20 hours, the cells were fixed and images (20x) were acquired with a confocal microscope. Bar: 50 µm. Arrows mark all the cells with mCherry-DAXX-HBD harbouring p62 bodies. The diameter of the biggest p62-GFP puncta (µm) in each cell was measured (LAS AF Lite). ***: P<0.0001. The number of p62-GFP puncta>0.5 µm in each cell was assessed (Image J). n=30, 14 cells. Statistical analysis was performed with T-tests. ***: P<0.0001. **(c)** DAXX overexpression increases p62-GFP puncta formation in HeLa Tet-on cells stably harbouring p62-GFP in "Tet-ON/OFF" conditions. HeLa Tet-on cells stably expressing p62-GFP were induced for expression for 24 hours. p62-GFP expression was then stopped by removing Dox, and mCherry vector or mCherry-DAXX was transfected into the cells. After 24 hours, the cells were fixed and images (20x) were acquired by a confocal microscope. Bar: 20 µm. The diameter of p62-GFP puncta were measured with Image J. n=88, 656 puncta. The diameters of all p62-GFP puncta or largest p62-GFP puncta (µm) in each cell, and the number of p62 puncta>0.5 µm were graphed. n=50, 34 cells. Statistical analysis was performed with T-tests. ***: P<0.0001. Data are shown as mean±sem in the figure. DX: DAXX.

Supplementary Figure 4

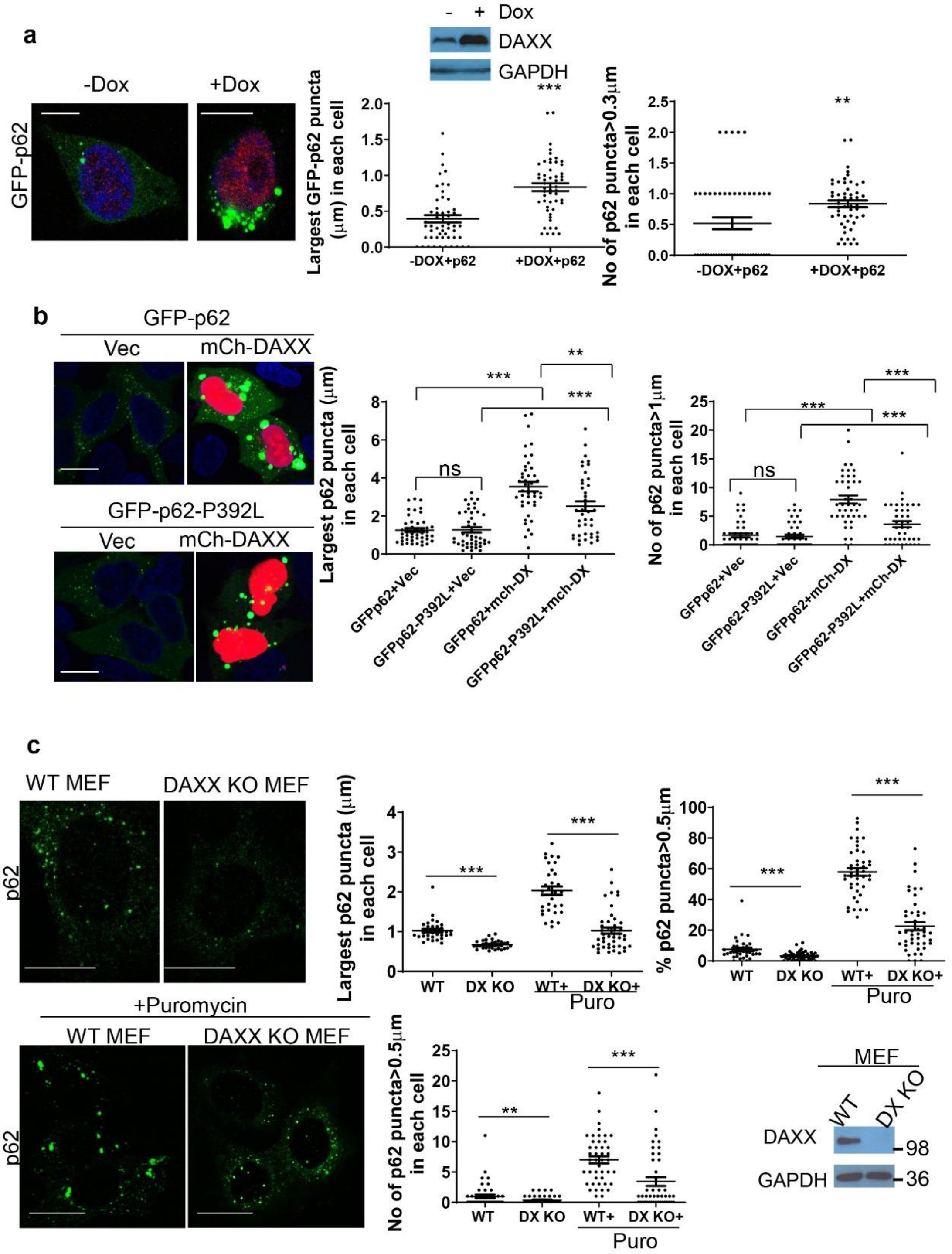

Supplementary Figure 4. DAXX promotes the formation of p62 bodies in multiple cellular models. (a) HeLa Tet-on cells stably expressing Dox-inducible DAXX were generated. GFP-p62 was transfected into the Dox-inducible DAXX stably expressing cells. GFP-p62 puncta were assessed in the cells in the presence or the absence of Dox. After 24 hours, the cells were fixed and were imaged by a confocal microscope. Bar: 10 μm. The diameter of GFP-p62 puncta was measured with Image J. The diameter of the largest GFP-p62 puncta (μm) in each cell, and the number of p62 puncta in each

cell>0.3 µm were plotted. n=52 cells. Statistical analysis was performed with T-tests. ***: P<0.0001 (left); **: P=0.0047 (right). Immunoblot shows that DAXX expression was induced by Dox. **(b)** GFP-p62 or GFP-p62 P392L was transfected into HeLa cells with empty vector or mCherry-DAXX, as indicated. After 20 hours, the cells were fixed. Images were acquired by a confocal microscope. Bar: 20 µm. The diameter of the largest GFP-p62 puncta (µm) in each cell was measured (Image J), and the number of p62 puncta>1 µm were graphed. n=45, 44, 42, 41 cells. Statistical analysis was performed with T-tests. ***: P<0.0001; **: P=0.0051; ns: P=0.9491 (left); ns: P=0.6549 (right). **(c)** WT and DAXX (DX) KO MEFs were treated without or with puromycin (10 µg/ml) for 6 hours, and the cells were stained with anti-p62. Images were acquired with a confocal microscope. Bar: 10 µm. The diameter of the biggest p62 puncta (µm) in each cell was measured; the percentage of p62 puncta>0.5 µm was assessed; the number of p62 puncta>0.5 µm in each cell was assessed (all with Image J). n=36, 50, 44, 45 cells. Statistical analysis was performed with T-tests. **: P=0.0041. ***: P=0.0003. Immunoblots show the expression of DAXX in Control or DAXX KO MEFs. Data are shown as mean±sem in the figure.

Supplementary Figure 5

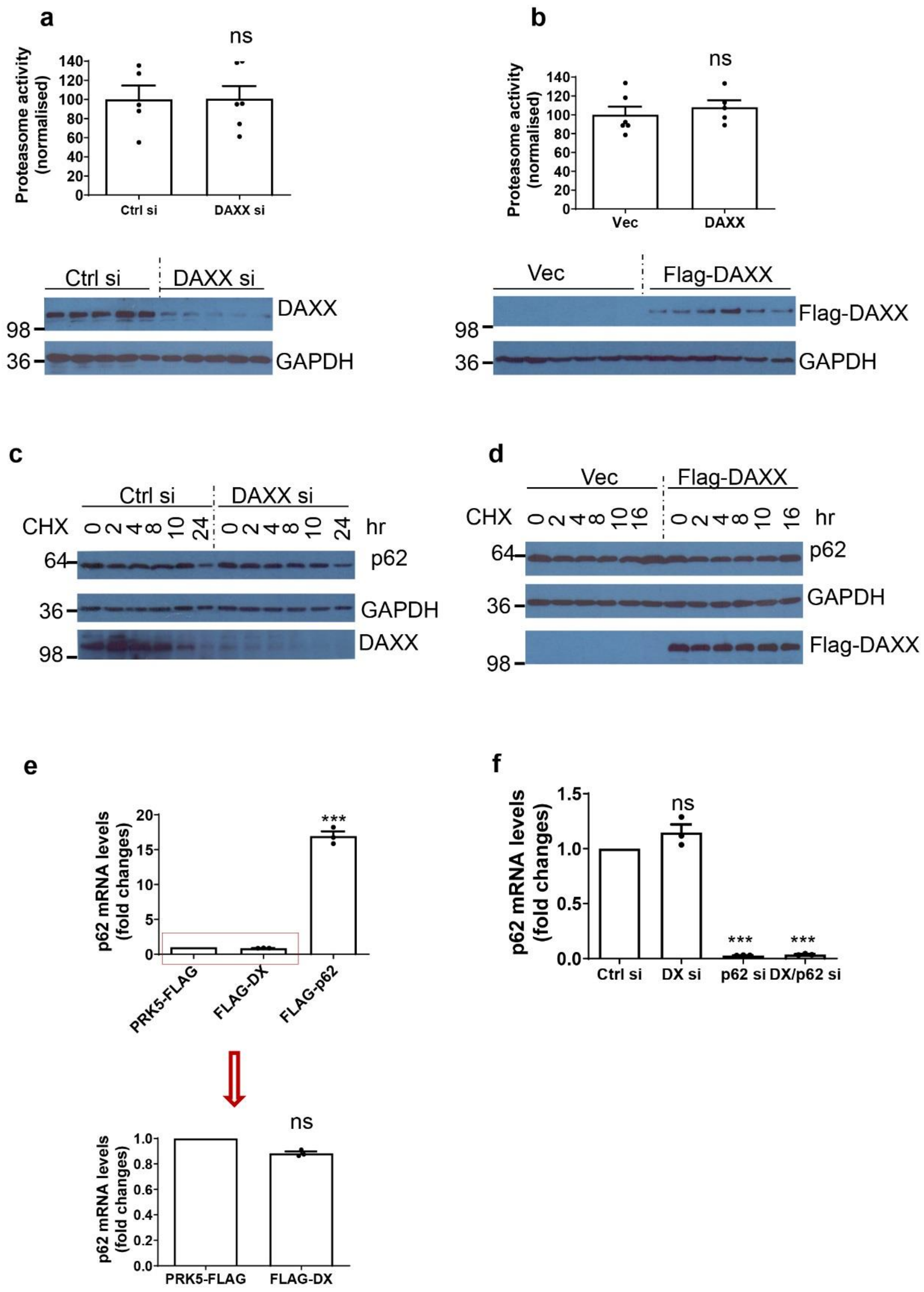

Supplementary Figure 5. DAXX does not significantly affect p62 protein expression levels. (a) HeLa cells were transfected with control or DAXX siRNA. After 48 hours, cells were harvested and subjected to proteasome assays. ns: P=0.9703. n=5, 6 biological replicates. A fraction of samples were subjected to immunoblot to check knockdown efficiency. **(b)** HeLa cells were transfected with control vector or Flag-

DAXX. After 24 hours, cells were harvested and subjected to proteasome assays. Data are shown as mean±sem. ns: P=0.5165. n=6 biological replicates. A fraction of samples were subjected to immunoblot with anti-Flag and GAPDH antibodies. **(c)** HeLa cells were transfected with control or DAXX siRNA. Cells were treated with cycloheximide (50 µg/ml) for indicated time. At 48 hours post-transfection, cells were harvested and subjected to immunoblot to anti-p62, anti-DAXX and GAPDH antibodies. **(d)** HeLa cells were transfected with control vector or Flag-DAXX. Cells were treated with cycloheximide (50 µg/ml) for indicated times. 48 hours post-transfection, cells were harvested and subjected to immunoblot with anti-p62, anti-Flag and GAPDH antibodies. **(e).** HeLa cells were transfected with control, Flag-DAXX, or Flag-p62 (as a positive control). After 24 hours, cells were harvested and subjected to RNA isolation**.** qPCR was performed with the isolated mRNAs. n=3 biological replicates (3 technical replicates) for each mRNA sample. Close-up view on the p62 mRNA levels of the boxed samples. Statistical analysis was performed by one-way ANOVA. Tukey's test was used for the comparison. ***: P<0.0001; ns: not significant. **(f).** HeLa cells were transfected with control, DAXX siRNA, p62 siRNA, or p62+DAXX siRNA. After 48 hours, cells were harvested and subjected to RNA isolation**.** qPCR was performed with the isolated mRNAs. n=3 biological replicates, and triplicate for each mRNA sample. Statistical analysis was performed by one-way ANOVA. Tukey's test was used for the comparison. ***: P<0.0001; ns: not significant. Data are mean±sem in the figure.

Supplementary Figure 6

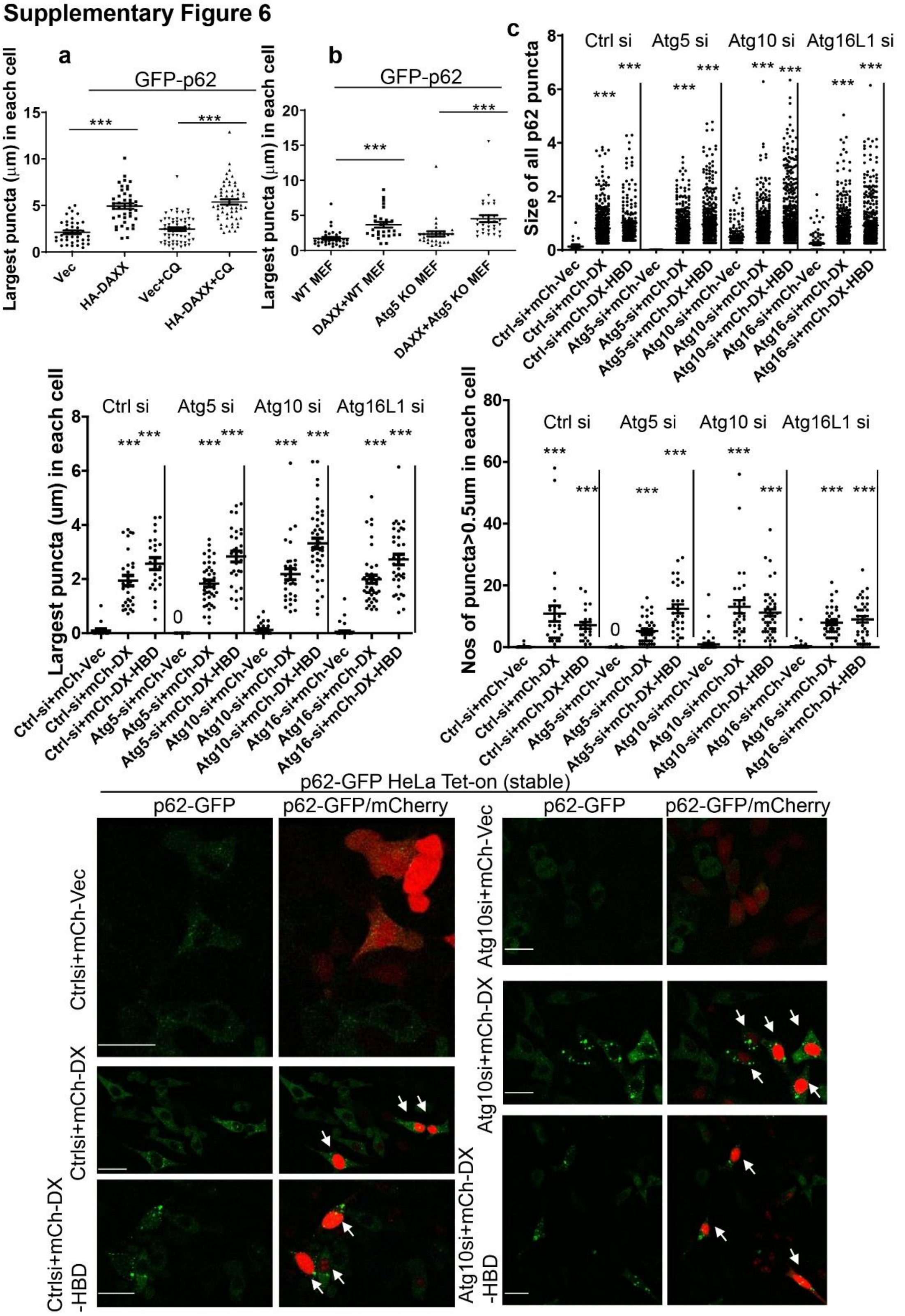

Supplementary Figure 6. DAXX promotes p62 body formation independently of autophagy. (a) GFP-p62 was co-transfected into HeLa cells with empty vector or HA-DAXX. After 3 hours, one set of transfected cells was treated with Chloroquine (CQ) (20 μM). After further 20 hours, the cells were fixed, and the size diameter of the biggest GFP-p62 puncta (μm) in each cell was measured (LAS AF Lite). n=40, 41, 59, 56 cells.

Statistical analyses were performed by T-tests. ***: P<0.0001 (both). **(b).** GFP-p62 was transfected into ATG5 WT or KO MEFs with empty vector or Myc-DAXX. After 20 hours, the cells were fixed and the size (diameter) of the biggest GFP-p62 puncta (μm) in each cell was measured (LAS AF Lite). n=34, 27, 31, 29 cells. Statistical analyses were performed by T-tests. ***: P<0.0001 (left); ***: P=0.0008 (right). **(c)** HeLa Tet-on cells harboring p62-GFP were transfected with control siRNA, Atg5 siRNA, Atg10 siRNA or Atg16L1 siRNA, respectively. The cells were split after 48 hours. 20 hours after the splitting, the cells were transfected with mCherry vector, mCherry-DAXX or mCherry-DAXX-HBD (178-417aa). 3 hours after the transfection, the cells were induced to express p62-GFP with Dox for 20 hours. The cells were fixed, and images were acquired with a confocal microscope. p62-GFP puncta were quantified as indicated. The diameters of all puncta (μm). n=18, 485, 198; n=31, 355, 502; n=136, 788, 658; n=72, 452, 452 puncta. The diameter of the largest puncta in each cell (μm), and the number of puncta>0.5 μm in each cell, were measured (Image J). n=16, 29, 23; n=31, 41, 30; n=29, 31, 44; n=53, 42, 36 cells. Statistical analysis was performed by one-way ANOVA. Tukey's test was used for the comparison. ***: P<0.0001. Knockdown efficiency was confirmed (Supplementary Figure 7b). Bar: 20 μm. Data are shown as mean±sem in the figure.

Supplementary Figure 7

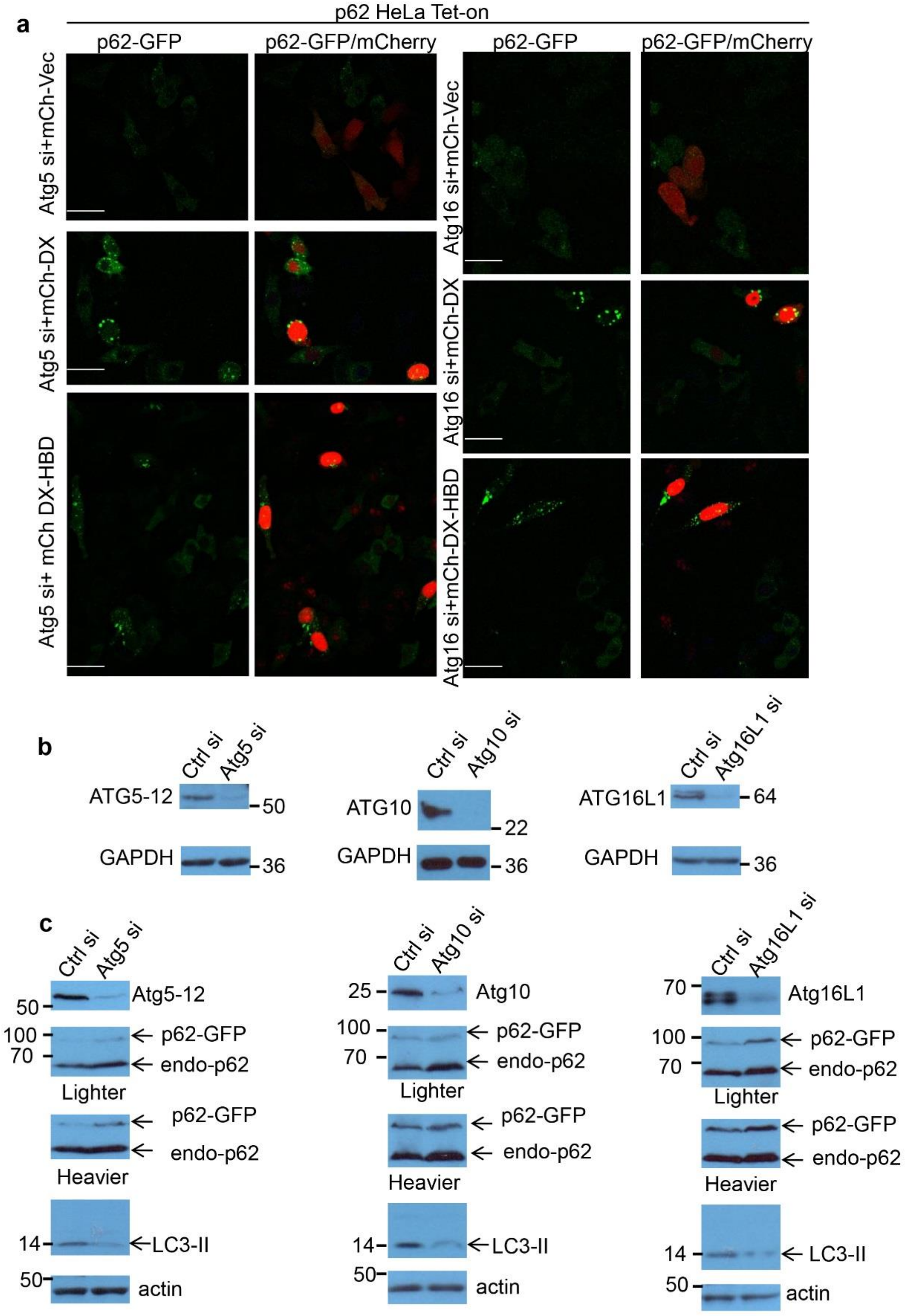

Supplementary Figure 7. DAXX promotes the formation of p62 bodies independently of autophagy. (a) HeLa Tet-on cells harboring p62-GFP were transfected with control siRNA, Atg5 siRNA or Atg16L1 siRNA, respectively. The cells were split after 48 hours. After 20 hours, the split cells were transfected with mCherry vector, mCherry-DAXX or mCherry DAXX (DX)-HBD (178-417aa). 3 hours after the

transfection, the cells were induced with Dox to express p62-GFP for 20 hours. The cells were then fixed, and images (20x) were acquired with a confocal microscope. Bar: 20 µm. **(b)** An aliquot of cells with each siRNA transfection were collected for immunoblot to confirm the knockdown efficiency. **(c).** Knockdown of autophagy machinery genes reduces LC3-II levels and increases p62 levels in p62-GFP expressing HeLa Tet-on cells. HeLa Tet-on cells harbouring p62-GFP were transfected with control siRNA, Atg5 siRNA or Atg16L1 siRNA, respectively. After 24 hours, the cells were induced with Dox to express p62-GFP for 24 hours. Cells were collected for immunoblot to confirm the knockdown efficiency. anti-Atg5, p62, LC3 or b-actin was used for the immunoblotting; anti-Atg10, p62, LC3 or b-actin was used for the immunoblotting; anti-Atg16L1, p62, LC3 or b-actin was used for the immunoblotting.

Supplementary Figure 8

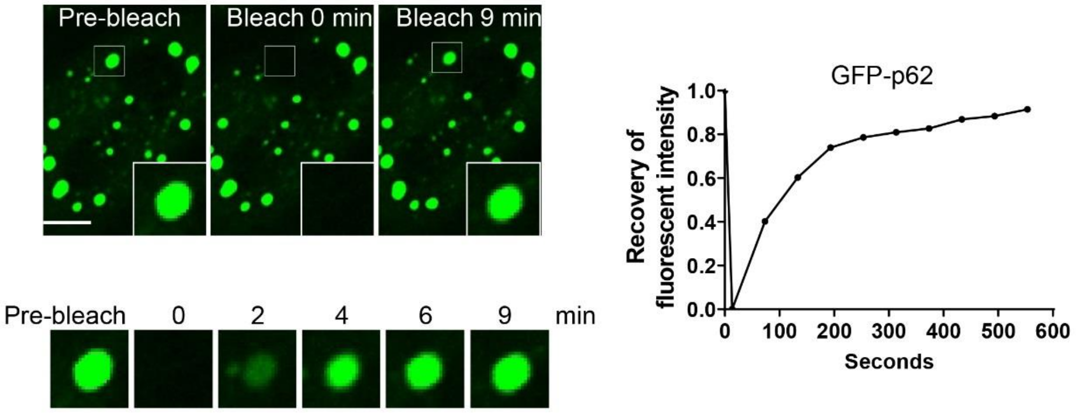

Supplementary Figure 8. Confirmation of p62 undergoing phase separation. HeLa cells were transfected with GFP-p62 for 24 hours, and subjected to FRAP (see the method for more details). The boxed punctum was subjected to bleaching. The images of pre-bleaching, bleaching and post-bleaching are shown. The right panel shows the relative fluorescence intensity of the GFP-p62 droplet at the bleaching time course. Bar: 10 µm.

Supplementary Figure 9

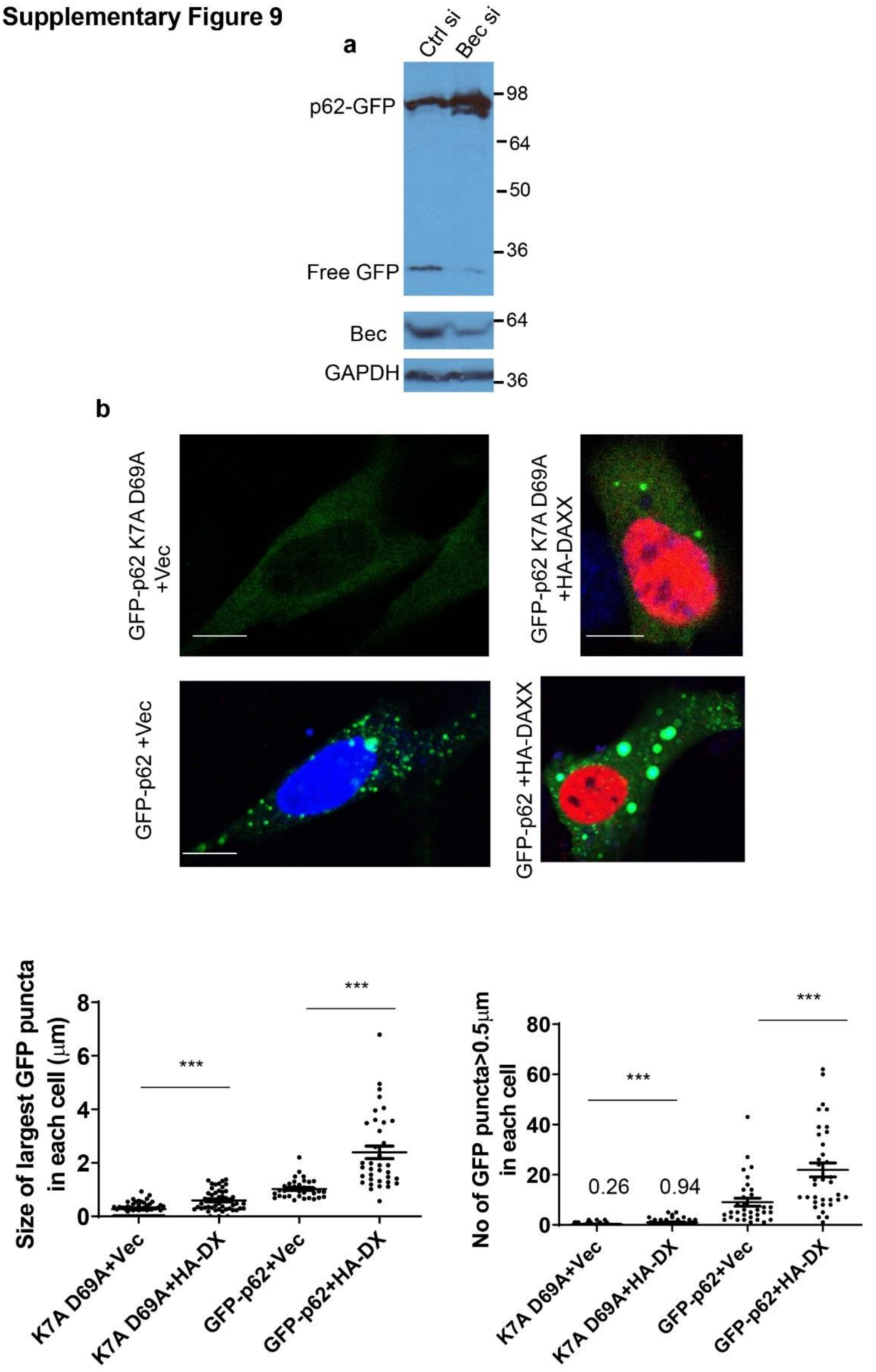

Supplementary Figure 9. DAXX promotes p62 oligomerisation. (a) p62-GFP stably expressing HeLa Tet-on cells were transfected with control or Beclin 1 siRNA. After 24 hours, Dox was added to induce p62-GFP expression. After another 24 hours, cells were harvested. The cell lysates were subjected to immunoblot with GFP, Beclin 1 and GAPDH antibodies. **(b)** GFP-p62 K7A D69A/vector, or GFP-p62 K7A D69A/HA-

DAXX were transfected into p62 knockout MEFs. In paralell, GFP-p62/vector, GFP-p62/HA-DAXX were also transfected into p62 knockout MEFs. After 20 hours, the cells were fixed, and subjected to HA antibody immunostaining. Images were acquired with a confocal microscope. Bar: 10 μm. The size of the largest GFP-p62 puncta in each cell was assessed (Image J). The number of GFP-p62 puncta>0.5 μm in each cell was assessed (Image J). The numbers (0.26; 0.94) represent the mean value of each column. n=53, 50, 34, 36 cells. Statistical analyses were performed by T-tests. ***: P<0.0001 (left); ***: P=0.0002, ***: P=0.0009 (right). Data are shown as mean±sem.

Supplementary Figure 10

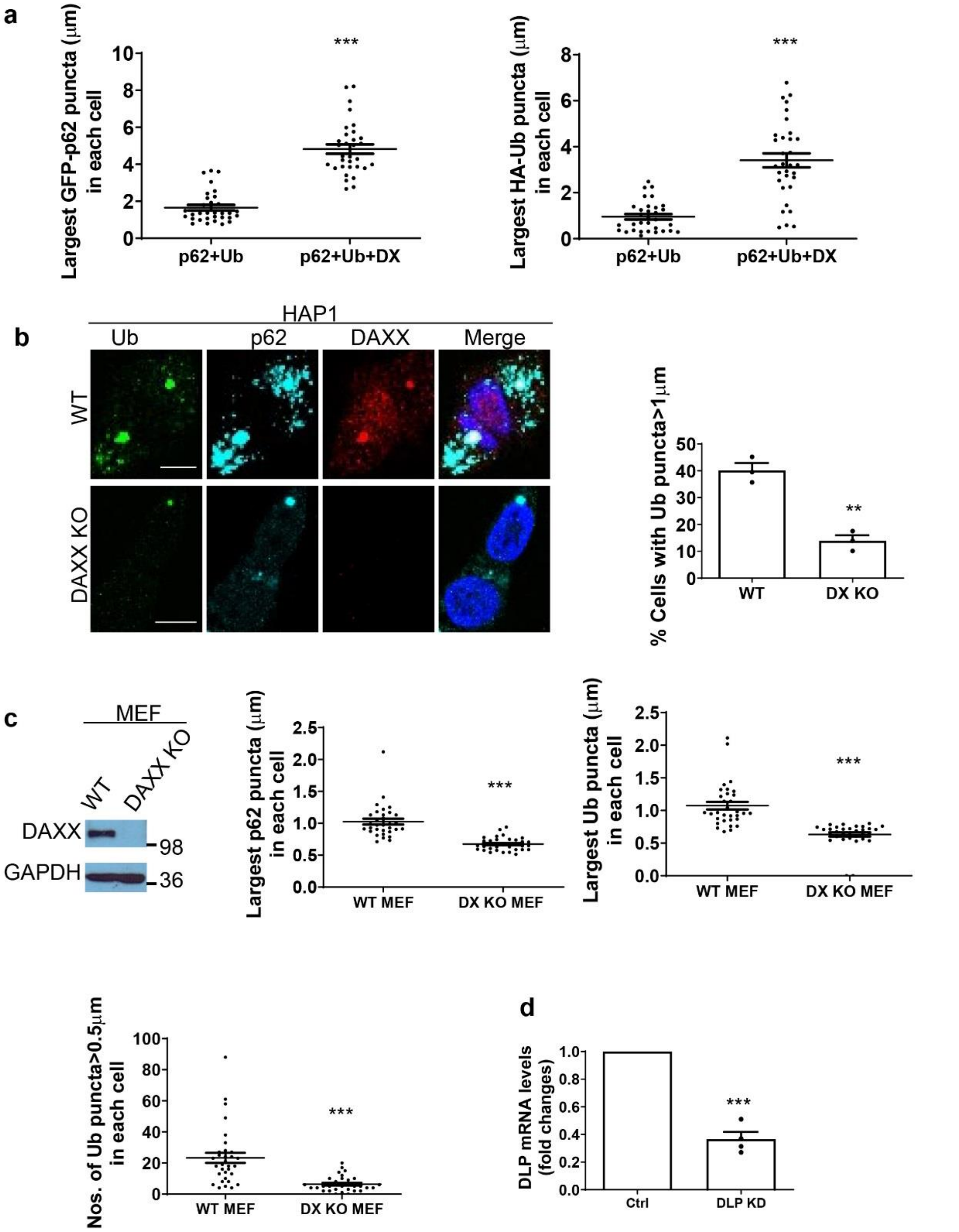

Supplementary Figure 10. DAXX promotes p62 colocalisation with polyubiquitinated proteins. (a) DAXX overexpression increases the sizes of p62 and ubiquitin puncta sizes. GFP-p62/HA-ubiquitin/vector or GFP-p62/HA-ubiquitin/Flag-DAXX were transfected into HeLa cells. After 20 hours, the cells were fixed and stained with anti-Flag and anti-HA antibodies (as described in **Fig. 7a)**. The diameter of the 4 largest GFP-p62 puncta and the diameter of the 4 largest HA-ubiquitin-positive foci were measured with LAS AF Lite in each cell. n=32 cells. Statistical analyses were performed by T-tests. ***: P<0.0001 (both). **(b)** Ubiquitin puncta sizes are decreased

in DAXX-KO HAP1 cells. WT HAP1 and DAXX (DX) KO HAP1 cells were treated with puromycin, and stained with anti-ubiquitin, anti-p62 and anti-DAXX antibody. The images were acquired by a confocal microscope. Bar: 5 µm. The diameter of the largest ubiquitin-positive puncta in each cell was measured with LAS AF Lite. The percentage of cells with ubiquitin puncta>1 µm was assessed. n=3 biological replicates. Statistical analyses were performed by T-tests. **: P=0.0093. **(c)** No DAXX expression in DAXX KO MEFs was confirmed by Western blot. The sizes of p62 foci and ubiquitin foci are reduced in DAXX KO MEFs. WT MEFs and DAXX KO MEFs were fixed and stained with p62 and ubiquitin antibody. The images were acquired by a confocal microscope. The diameter of the largest p62 puncta and ubiquitin puncta in each cell was quantified with Image J. The number of ubiquitin puncta >0.5 µm in each cell was assessed. n=32 cells. Statistical analyses were performed by T-tests. ***: P<0.0001 (all). **(d)** qPCR was performed with the mRNAs from dissected brains of control or DLP (DAXX) knockdown flies. n=4 biological replicates (3 technical replicates) for each mRNA sample. Statistical analyses were performed by T-test. ***: P=0.0009. Data are mean±sem in the figure.

Supplementary Figure 11

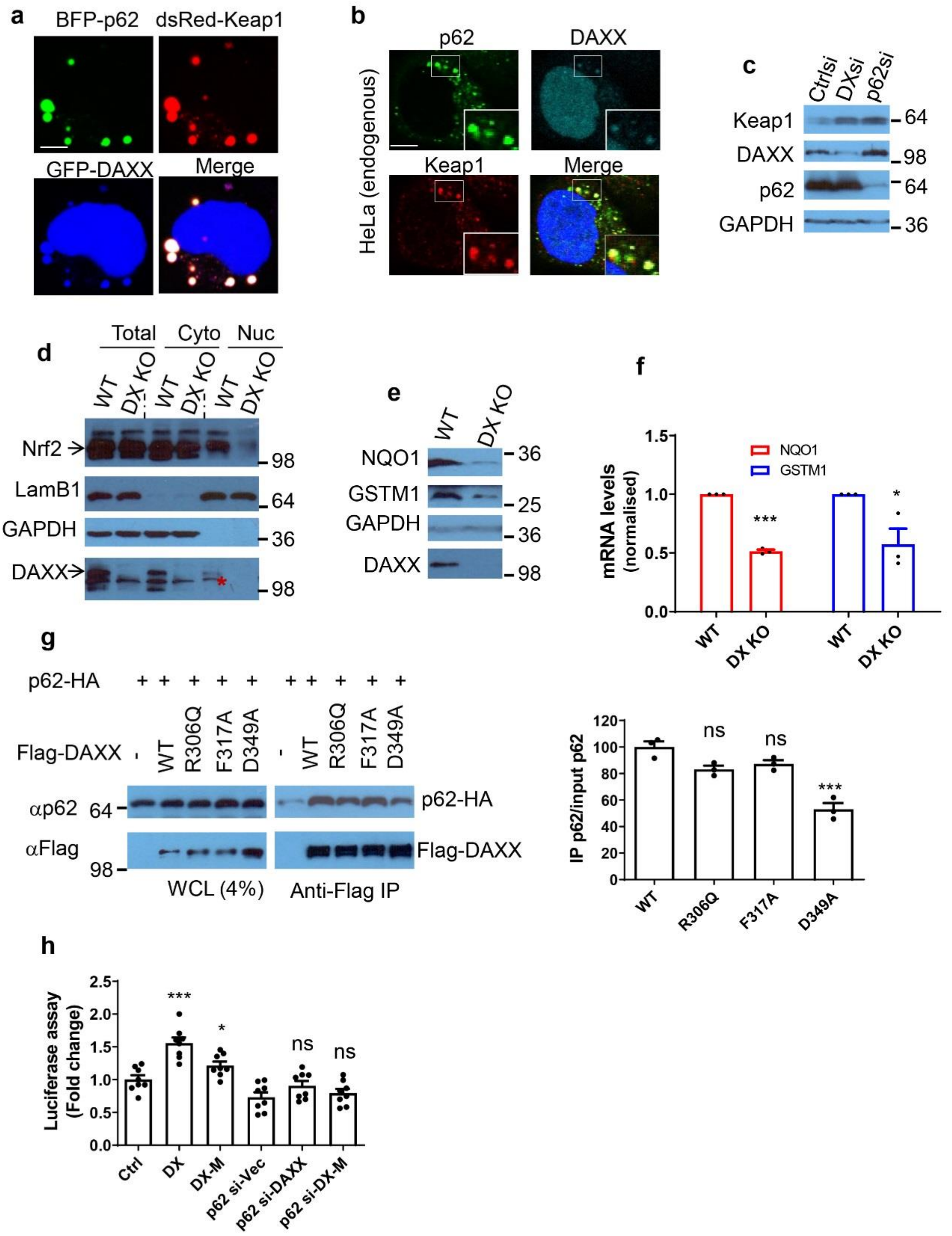

Supplementary Figure 11. DAXX activates Nrf2-mediated stress response. (a) BFP-p62, dsRed-Keap1 and GFP-DAXX were co-transfected into HeLa cells. After 20 hours, cells were fixed, and imaged with confocal microscopy. Bar: 10 µm. **(b)** HeLa cells were fixed and stained with anti-p62, anti-Keap1, and anti-DAXX; cells were imaged with confocal microscopy. Bar: 10 µm. **(c)** HeLa cells were knocked down with siRNAs as indicated for 48 hours. Cells were lysed and subjected to immunoblot with indicated antibodies. **(d).** WT or DAXX KO HAP1 cells were subjected to fractionation

for cytoplasmic and nuclear fractions. The total lysates, cytoplasmic (Cyto) or nuclear (Nuc) fractions were used for immunoblot with anti-Nrf2, Lamin B1 (LamB1), DAXX, or GAPDH antibody. The red star denotes the remaining Nrf2 signals from the previous blot. Lamin B1: nuclear marker. **(e)** WT or DAXX KO HAP1 cells were lysed and subjected to immunoblot with indicated antibodies. **(f)** qPCR was performed with mRNAs from WT or DAXX KO HAP1 cells. n=3 biological replicates (3 technical replicates). Statistical analyses were performed by T-tests. ***: P=0.0008; *: P=0.0444. **(g)** p62-HA/empty vector, p62-HA/Flag-DAXX, or p62-HA/Flag-DAXX mutants as indicated were cotransfected into HeLa cells. After 20 hours, anti-Flag was used for immunoprecipitation (IP). The immunoprecipitates and the whole cell lysates (WCL) were probed with anti-p62, and anti-Flag. The ratios of immunoprecipitated p62 versus input p62 were quantified. Statistical analysis was performed by one-way ANOVA. Tukey's test was used for the comparison. n=3 independent experiments. ***: P<0.0001. ns: not significant. **(h)** HeLa cells were transfected with empty vector, wild-type DAXX (DX) or DAXX D349A mutant (DX-M) along with with luciferase reporter components. In parallel, HeLa cells were knocked down with p62 siRNA, and subsequently transfected with empty vector, DX or DX-M along with luciferase reporter components (as described in the method). After 20 hours, cell lysates were subjected to dual luciferase assays (see methods). Statistical analysis was performed by one-way ANOVA. Tukey's test was used for the comparison. n=8 biological replicates. ***: P<0.0001; *: P<0.05; ns: not significant. Data are shown as mean±sem in the figure.

Supplementary Figure 12

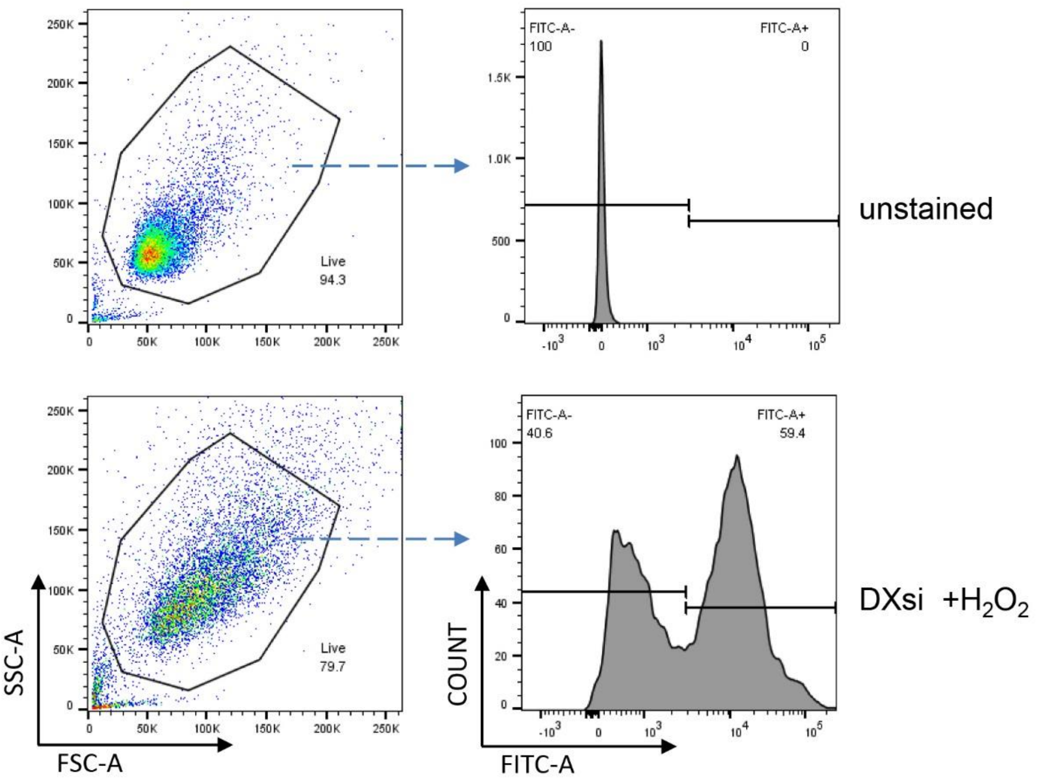

Supplementary Figure 12. Gating strategies used for flow cytometry. HeLa cells were transfected with siRNAs, treated with control or H2O2, and collected for H2DCFDA (DCF) staining. Representative examples are showed. In the preliminary FSC/SSC gating, main population was gated to exclude debris. The main population was then subjected to ROS assay. Gating for Fig. 8d-e was performed with the same standard as shown in the figure.

Supplementary Figure 13 Full scans of uncropped blots

Fig. 1a

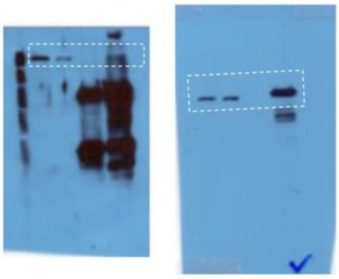

Fig. 1b

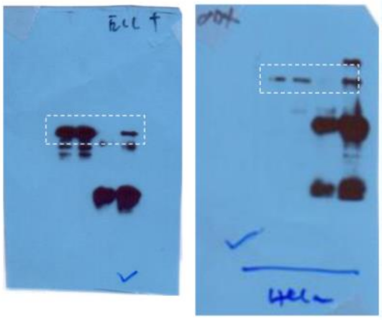

Fig. 1c

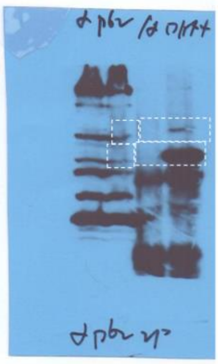

Fig. 1h

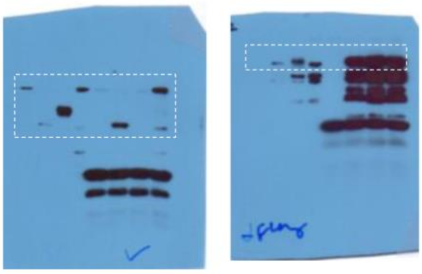

Fig. 3e

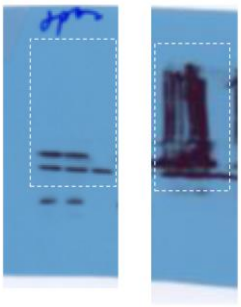

Fig. 4e

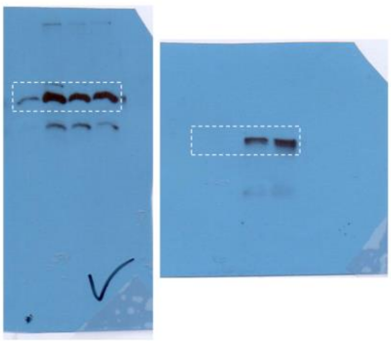

Fig. 5a

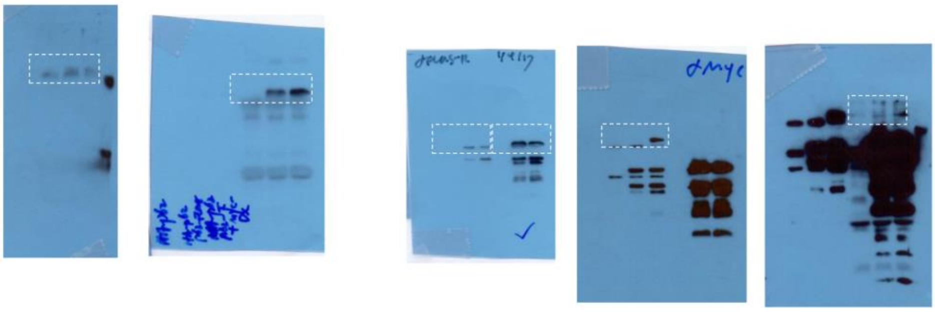

Supplementary Figure 13-continued Full scans of uncropped blots

Fig. 5b

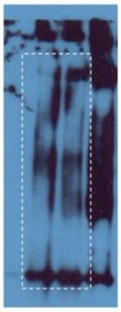

Fig. 5c

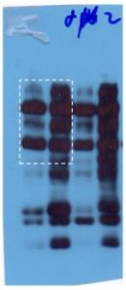

Fig. 5e

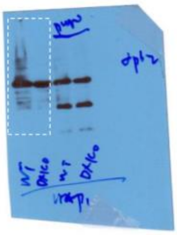

Fig. 5f

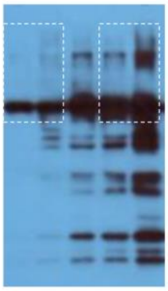

Fig. 5g

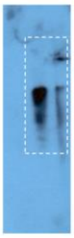

Fig. 6b

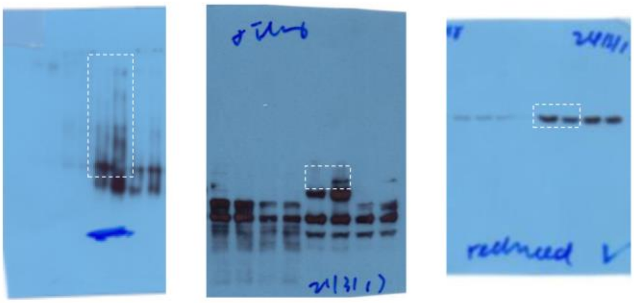

Fig. 6c

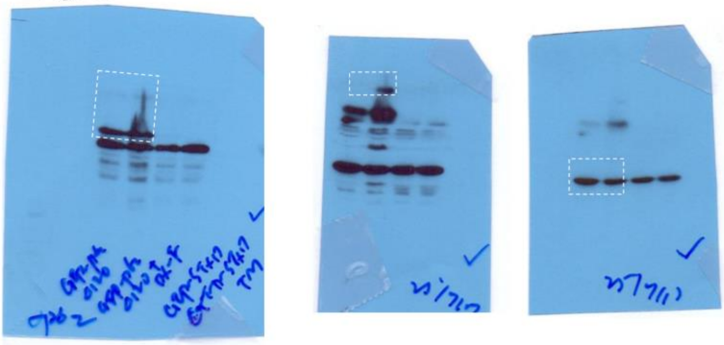

Fig. 7d

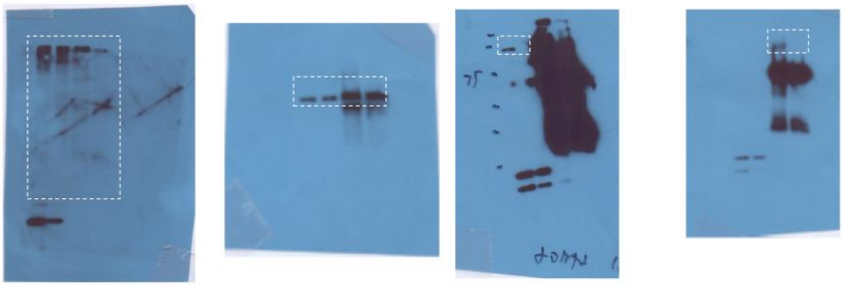

Supplementary Figure 13-continued Full scans of uncropped blots

Fig. 7e

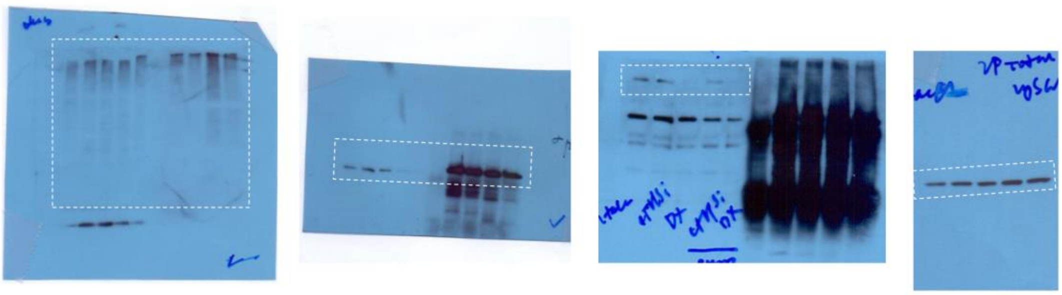

Fig. 8b

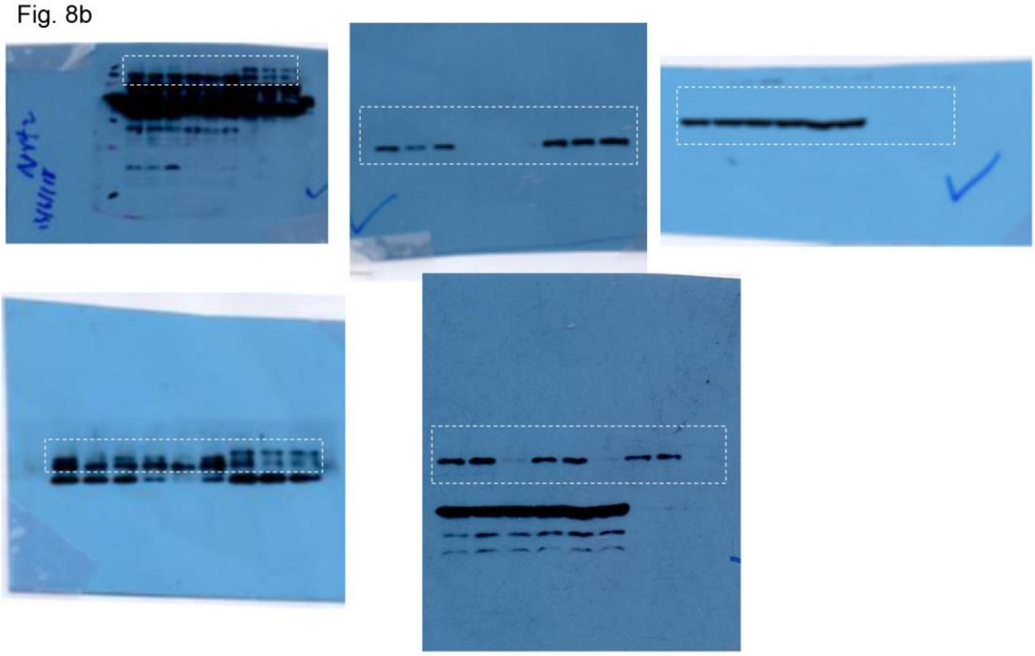

Fig. 8c

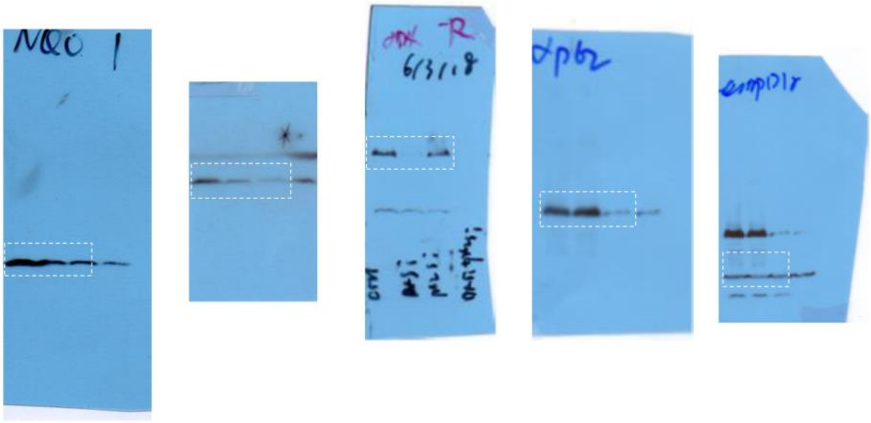

Supplementary Table 1

Plasmids generated in the study	Vectors (cut sites 5'/3')	Inserts (cut sites 5'/3')
pEGFP-N3-p62	pEGFP-N3 (BglII/EcoRI)	p62 (BglII/EcoRI)
pTRE-2Hyg-p62-GFP	TRE-2Hyg (BamHI/NotI)	p62-GFP (BglII/NotI from pEGFP-N3-p62)
pCMV6m-Myc-DAXX	pCMV6M (BamHI/EcoRI)	DAXX (BamHI/EcoRI)
pCMV6m-Myc-DAXX-N	pCMV6M (BamHI/EcoRI)	DAXX1-370aa (BamHI/EcoRI)
pCMV6m-Myc-DAXX-C	pCMV6M (BamHI/EcoRI)	DAXX(371-740aa) (BamHI/EcoRI)
pEGFP-C1-p62	pEGFP-C1 (BamHI/EcoRI)	p62 (BglII/EcoRI)
pEGFP-C1 p62-del120 (PB1)	pEGFP-C1 (BglII/EcoRI)	p62-del PB1 (BglII/EcoRI)
pcDNA3-mCherry	pcDNA3 (KpnI/BamHI)	mCherry (KpnI/BamHI)
pcDNA3-mCherry-DAXX	pcDNA-mCherry (BamHI/EcoRI)	DAXX (BamHI/EcoRI)
pcDNA3-mCherry-DAXX-N	pcDNA-mCherry (BamHI/EcoRI)	DAXX1-370aa (BamHI/EcoRI)
pcDNA3-mCherry-DAXX-C	pcDNA-mCherry (BamHI/EcoRI)	DAXX371-740aa (BamHI/EcoRI)
pcDNA3-mCherry-DAXX-178-417	pcDNA-mCherry (BamHI/EcoRI)	DAXX178-417aa (BamHI/EcoRI)
pCE-BiFC-VC155-DAXX	BiFC-155 (EcoRI/KpnI)	DAXX (EcoRI/KpnI)
pCE-BiFC-VN173-p62	BiFC-173 (EcoRI/XbaI)	p62 (EcoRI/XbaI)
pGADT7-DAXX-370 (1-370)	pGADT7 (EcoRI/BamHI)	DAXX1-370aa (EcoRI/BamHI)
pGBKT7-p62-385 (1-385)	pGBKT7-p62-385 (EcoRI/BamHI)	p62 1-385aa (EcoRI/BamHI)
pcDNA4/TO-p62-HA	HA-vector (EcoRI/NotI)	p62 (EcoRI/NotI)
pcDNA4/TO-p62-385-HA	HA-vector (EcoRI/NotI)	p62 1-385aa (EcoRI/NotI)
pcDNA4/TO-p62-185-HA	HA-vector (EcoRI/NotI)	p62 1-185aa (EcoRI/NotI)
pcDNA4/TO-p62-120-HA	HA-vector (EcoRI/NotI)	p62 1-120aa (EcoRI/NotI)
pcDNA4/TO-p62-245-HA	HA-vector (EcoRI/NotI)	p62 1-245aa (EcoRI/NotI)
pcDNA4/TO-p62-300-HA	HA-vector (EcoRI/NotI)	p62 1-300aa (EcoRI/NotI)
pcCMV5c-p62-Flag	pCMV-5c (EcoRI/NotI)	p62 (EcoRI/NotI)
pCMV6M-Myc-DAXX-150	pCMV Myc (BamHI/NotI)	DAXX 1-150aa (BamHI/NotI)
pCMV6M-Myc-DAXX-182	pCMV6M (BamHI/NotI)	DAXX 1-182aa (BamHI/NotI)
pCMV6M-Myc-DAXX-200	pCMV6M (BamHI/NotI)	DAXX 1-200aa (BamHI/NotI)
pCMV6M-Myc-DAXX-230	pCMV6M (BamHI/NotI)	DAXX 1-230aa (BamHI/NotI)

Supplementary Table 1

pCMV6M-Myc-DAXX-del 150	pCMV Myc (BamHI/NotI)	DAXX del 1-150aa (BamHI/NotI)
pCMV6M-Myc-DAXX-del 160	pCMV Myc (BamHI/NotI)	DAXX del 1-160aa (BamHI/NotI)
pCMV6M-Myc-DAXX-del 170	pCMV Myc (BamHI/NotI)	DAXX del 1-170aa (BamHI/NotI)
pCMV6M-Myc-DAXX-del 190	pCMV Myc (BamHI/NotI)	DAXX del 1-190aa (BamHI/NotI)
pCMV6M-Myc-DAXX-del 230	pCMV Myc (BamHI/NotI)	DAXX del 1-230aa (BamHI/NotI)
pGEX-6P1-GST-p62-del PB1 (bacterial)	pGEX-6P1 (BglII/EcoRI)	p62-del PB1 (BglII/EcoRI)
pGEX-6P1-GST-DAXX-1-250 (bacterial)	pGEX-6P1 (BamHI/EcoRI)	DAXX 1-250aa (BamHI/EcoRI)
pEBG-GST-Bad	pEBG-GST vector (BamH1/NotI)	Bad (BamH1/NotI)
pEBG-GST-LC3A	pEBG-GST vector (BamH1/NotI)	LC3A (BamH1/NotI)
pcDNA3-mCherry-LC3B	pcDNA3 mCherry (BamHI/NotI)	LC3B (BamHI/NotI)
pcDNA3-p62 (*in vitro* translation)	pcDNA3 (BamHI/EcoRI)	p62 (BglII/EcoRI)
pcDNA3-DAXX (*in vitro* translation)	pcDNA3 (BamHI/NotI)	DAXX (BamHI/NotI)
pET28a-p62	pET28a (BamHI/Not I)	p62 (BglII/NotI)
pET28a-DAXX	pET28a (BamHI/Not I)	DAXX (BamHI/NotI)
pTRE-2hyg-DAXX	pTRE-2hyg (BamHI/NotI)	DAXX (BamHI/NotI)

Supplementary Table 2

Bait	Preys HGNC symbols	Prey gene descriptions
SQSTM1/p62	ACSS3	acyl-CoA synthetase short-chain family member 3
SQSTM1/p62	AHNAK	AHNAK nucleoprotein
SQSTM1/p62	AJUBA	ajuba LIM protein
SQSTM1/p62	BAIAP2	BAI1 associated protein 2
SQSTM1/p62	CDC42	cell division cycle 42
SQSTM1/p62	DAXX	death domain associated protein
SQSTM1/p62	DCAF8	DDB1 and CUL4 associated factor 8
SQSTM1/p62	DYNLT1	dynein light chain Tctex-type 1
SQSTM1/p62	FABP7	fatty acid binding protein 7
SQSTM1/p62	FAM131A	family with sequence similarity 131 member A
SQSTM1/p62	FANCG	Fanconi anemia complementation group G
SQSTM1/p62	FYN	FYN proto-oncogene, Src family tyrosine kinase
SQSTM1/p62	HDAC6	histone deacetylase 6
SQSTM1/p62	LCK	LCK proto-oncogene, Src family tyrosine kinase
SQSTM1/p62	MAP2K5	mitogen-activated protein kinase kinase 5
SQSTM1/p62	MAPK8	Mitogen-activated protein kinase 8
SQSTM1/p62	MLST8	MTOR associated protein, LST8 homolog
SQSTM1/p62	MRFAP1	Mof4 family associated protein 1
SQSTM1/p62	NLK	nemo like kinase
SQSTM1/p62	POLR2E	RNA polymerase II subunit E
SQSTM1/p62	PRKCZ	protein kinase C zeta
SQSTM1/p62	RAC1	rho family, small GTP binding protein Rac1
SQSTM1/p62	RIPK1	receptor interacting serine/threonine kinase 1
SQSTM1/p62	RNF2	ring finger protein 2
SQSTM1/p62	SDC1	syndecan 1
SQSTM1/p62	SERBP1	SERPINEE1 mRNA binding protein 1
SQSTM1/p62	SOD1	superoxide dismutase 1
SQSTM1/p62	STUB1	STIP1 homology and U-box containing protein 1
SQSTM1/p62	TCP1	t-complex 1
SQSTM1/p62	TP53	tumor protein p53
SQSTM1/p62	TRAF6	TNF receptor associated factor 6
SQSTM1/p62	UBC	ubiquitin C
SQSTM1/p62	ULK1	unc-51 like autophagy activating kinase 1
SQSTM1/p62	VHL	von Hippel-Lindau tumor suppressor
SQSTM1/p62	WASL	Wiskott-Aldrich syndrome like
SQSTM1/p62	WDR61	WD repeat domain 61
SQSTM1/p62	ZFAND5	zinc finger AN1-type containing 5
SQSTM1/p62	ZNF767	zinc finger family member 767
